# The impact of the COVID-19 pandemic on the career aspirations of adolescents in Romania: a longitudinal analysis

**DOI:** 10.3389/fsoc.2025.1653577

**Published:** 2025-12-03

**Authors:** Adrian Hatos, Delia-Georgeta Bekesi, Mirela Lăcrimioara Cosma, Zsolt-Botond Bottyan, Denisa Dobai, Simina-Maria Miko

**Affiliations:** 1 Department of Sociology and Social Work, University of Oradea, Oradea, Romania; 2 Department of Sciences of Education, University of Oradea, Oradea, Romania; 3 County Center for Educational Resources and Assistance-Bihor, Oradea, Romania

**Keywords:** adolescents, career optimism, occupational aspirations, COVID-19 pandemic, longitudinal research, human capital theory

## Abstract

**Introduction:**

This study explores the evolving landscape of career aspirations among Romanian eighth-grade students by analyzing survey data collected both before and during the COVID-19 pandemic. Eighth-grade students were chosen for this study because they are at a critical educational crossroads, highly influenced by socio-economic and emotional factors, and their career aspirations, which evolved during the pandemic’s effects on education, social interaction, and economic uncertainty, can be tracked over time for a longitudinal analysis. The research has three main objectives: first, to assess whether during the pandemic career uncertainty increased, foundational career optimism (defined by educational commitment) and strategic career planning (defined by mobility intentions) of Romanian adolescents were modified during the pandemic; second, to identify shifts in the perceived status of desired occupations using the International Socio-Economic Index of Occupational Status (ISEI) and Standard International Occupational Prestige Scale (SIOPS); and third, to examine changes in students’ intentions to study or work abroad. The first wave (n= 1 643) was conducted before the pandemic (December 2018–January 2019), while the second (May–June 2021, *n* = 922) and third wave (January–February 2022, *n* = 1217) took place during the pandemic.

**Methods:**

To test the research hypotheses and address the limitations of existing studies, we adopted a longitudinal design. Specifically, we compared data from three waves of the MERPAS survey (Educational Monitor of Results, Practices, and Attitudes in Schools of Bihor), conducted among eighth-grade students (aged 13–15) in the Bihor County of Romania.

**Results:**

The findings reveal an improvement in students’ clarity regarding their career goals, as indicated by a higher proportion of valid responses about their desired professions. Additionally, the average occupational status, measured by the ISEI and SIOPS scales, showed a slight but statistically significant increase during the pandemic. The data also indicates a notable decline in students’ intentions to pursue educational or professional opportunities abroad.

**Discussion:**

The findings suggest that Romanian adolescents, unlike peers in other international studies, did not experience a decline in career optimism or an increase in uncertainty. Instead, the pandemic appears to have prompted a rational reassessment of career trajectories. Framed by human capital theory, the results indicate that adolescents responded to the “career shock” of the pandemic by increasing their investment in “safe” high-status professions requiring higher education (hence the rise in ISEI/SIOPS scores) while simultaneously reducing exposure to the health and logistical risks of international mobility. This highlights the critical role of local socio-economic context in mediating the pandemic’s impact on youth career aspirations.

## 1 Introduction and literature review

### 1.1 Broad context

The COVID-19 pandemic profoundly impacted societies worldwide, reshaping economies, public health, and youth career trajectories, with international studies reporting declining career optimism and increasing uncertainty among adolescents (Hussong et al., 2021; Carey et al., 2023). Economically, Romania demonstrated remarkable resilience with a milder GDP contraction in 2020 (−3.7%) compared to the European Union average (−6.1%) and steeper declines in Italy (−9.6%) and France (−8.9%) (Siegel, 2024), attributed to less restrictive lockdowns in industry and construction, along with a stable agricultural sector, with Bihor County specifically benefiting from government interventions despite export disruptions (Negrea and Bekesi, 2020), followed by robust recovery with GDP rebounding by 5.9% in 2021, surpassing the EU’s 5.3% growth, and continued expansion in 2022 (4.8% vs. the EU’s 3.4%), supported by financial aid and NRRP investments (Union, E, 2024; Eurostat, 2024). However, Romania experienced severe social impacts, including an excess mortality rate of 30.3% in September 2021—one of the highest in the EU (Chirtoc, 2021)—and life expectancy dropping to 72.8 years, reversing 14 years of progress (Stiftung, 2023), while education was significantly disrupted with schools remaining fully closed for 108 days and partially closed for another 49 days—one of the longest shutdowns in Europe compared to the UNECE average of 13 weeks (UNICEF, 2021; UNECE, 2022)—resulting in approximately 1.5 years of educational disruption and severe learning losses that surpassed those in countries like the Netherlands, where disruptions lasted only a few months (Shmis et al., 2020; Romania-Insider, 2022; Rajasekaran and Casap, 2022). This study examines the career aspirations of Romanian eighth graders within this unique context of economic resilience coupled with severe educational and social disruptions, offering valuable insights into how these macroeconomic and social factors influenced youth career trajectories.

### 1.2 Romanian adolescents’ career aspirations

Romanian adolescents demonstrate complex patterns in career development, with research revealing that gender, maternal occupational status as measured by the International Socio-Economic Index (ISEI), ethnicity, and academic achievements significantly shape their professional aspirations, though students belonging to the Hungarian ethnic minority exhibit lower levels of career clarity compared to their Romanian counterparts (Mikó and Hatos, 2024). While occupational aspirations appear relatively stable over time, providing reasonable validity for career choice responses, Romanian youth face a substantial migration challenge, as almost half of young people aged between 16 and 35 express a desire to emigrate (Cristea et al., 2021), with younger individuals showing higher emigration probabilities (Roman and Vasilescu, 2016). This brain drain phenomenon (Iacob, 2018) is particularly pronounced among technical university students, where economic factors serve as the primary migration driver, potentially resulting in Romania losing up to 40% of its population by 2060 due to outward migration (Gherheș et al., 2020). The convergence of these factors creates a scenario where Romanian adolescents maintain ambitious career aspirations influenced by socioeconomic background, while simultaneously considering international opportunities for better economic prospects and living standards (Petru-Cristian, 2024; Ion et al., 2022; Mikó and Hatos, 2024).

The pandemic potentially reshaped adolescents’ career aspirations, occupational status perceptions, and international mobility intentions (Hatos and Gyarmati, 2023), yet little is known about how these evolved over time in this specific context. The pandemic’s multifaceted effects—including economic uncertainty, shifts in labor markets, changes in educational delivery, and altered social dynamics—likely created complex changes in how young people envision their professional futures and geographic mobility. However, little empirical evidence exists examining how these critical developmental outcomes evolved during this unprecedented period.

### 1.3 COVID-19 pandemic impacts

The COVID-19 pandemic significantly impacted adolescents’ well-being, social interactions, education, and career-related decisions. Studies reported decreased positive affect and increased negative affect among teens during this period (Cockerham et al., 2021), with heightened levels of anxiety, depression, and loneliness linked to prolonged social isolation (Bemme et al., 2021; Pierce et al., 2025). Research on Romanian adolescents by Maftei et al. (2022) showed that most children and adolescents associated masks with the coronavirus, predominantly evoking sadness (45.2%) and fear (17.4%), while online schooling was perceived negatively due to poor interaction and disruption of regular routines. Similarly, a study conducted on Romanian adolescents by Mareş et al. (2022) found that the pandemic and online schooling influenced students’ career decisions, generating confusion and indecision, especially among disadvantaged groups, as limited access to job shadowing and orientation visits reduced exposure to reliable career information sources. Despite a strong desire for self-determination, disadvantaged students often relied on family, particularly parents (32.7%), and professionals in the field of interest (45.8%) for guidance, while formal education remained a key factor in career decision-making. Importantly, although Maftei et al. (2022) identified positive thoughts and strengthened family relationships as coping strategies, more than half of their participants perceived no positive outcomes of the pandemic, while 40% emphasized increased family time as beneficial. By contrast, Mareş et al. (2022) highlighted that many disadvantaged Romanian adolescents maintained an optimistic outlook on their professional future, with 43% strongly believing in their creative potential and 35% envisioning future success despite the crisis. Complementing these findings, international research showed increased time spent alone, often on computer games, social media, and television (Kalenkoski and Pabilonia, 2025), and widespread dissatisfaction with remote education, which raised concerns about school dropout and inequalities, particularly affecting marginalized groups (Ramaiya et al., 2023). Overall, these results emphasize that while the pandemic exacerbated psychosocial difficulties and educational disparities, it also revealed adolescents’ resilience and self-determination in navigating personal, academic, and career-related challenges, underscoring the need for targeted interventions that prioritize mental health, career guidance, and equitable educational opportunities in post-pandemic recovery efforts (Pierce et al., 2025; Ramaiya et al., 2023).

### 1.4 Research problem and objectives

In the research literature, career aspirations are broadly defined as the ambitions, expectations, and desired conditions an individual holds for their future professional life. In our research, we focus on the social status, or prestige, of occupational aspirations of youth, as a variant of quantitatively describing career aspirations, measured as the status of profession one desires to have at 30 using well-established scales of occupational status (SIOPS).

The COVID-19 pandemic generated an unprecedented surge in research on youth career aspirations, both educational and professional, explored through diverse designs ranging from cross-sectional studies (Akbari and Faisal, 2023; Ashta et al., 2023; Yarde et al., 2022) and longitudinal approaches (Baskar et al., 2022; Glick et al., 2025; von Soest et al., 2022), to quantitative and qualitative investigations (Desrochers et al., 2020; Henkens et al., 2022), employing methods such as direct survey questions on educational aspirations as proxies for career expectations (Glick et al., 2025), Likert scales and factor analysis to assess optimism (Fakkel et al., 2023), occupational certainty and ambition indicators across multinational datasets (Mann et al., 2020), stress, and self-efficacy measures including the CDMSE-SF (*Career Decision-Making Self-Efficacy—Short Form*) (Ashta et al., 2023; Wahyuningsih et al., 2023), retrospective questions despite reliability concerns (Akbari and Faisal, 2023; Baskar et al., 2022; Cournoyer and Rohner, 1996; Hatos and Gyarmati, 2023), and field-specific surveys such as Strohmeier et al. (2024) on digital professions, Tran et al. (2020) on STEM-oriented students, and Zhang et al. (2022) who documented a rise in interest in medical careers from 17.5 to 29.6% among Chinese adolescents.

Although several articles conclude that aspirations declined, few refer to quantitative measures of aspirations beyond educational aspirations. While occupational status is one of the most common measures of occupational aspiration, such measures were rarely used in the reviewed literature. Although some articles refer to changes in perspective concerning specific job categories—such as STEM or medical professions—few approach the entire spectrum of occupations from which students can choose.

The current study addresses these gaps by utilizing longitudinal data to examine changes in career certainty, occupational status aspirations measured through established indices (ISEI, SIOPS), and international mobility intentions among Romanian adolescents, comparing pre-pandemic and during-pandemic periods in a context characterized by unique economic resilience coupled with severe educational disruptions. Therefore, the objective of this study is to examine changes in adolescents’ career aspirations, occupational status perceptions, and international mobility intentions by comparing pre-pandemic and during-pandemic data, with findings offering important insights for understanding post-pandemic youth development and informing educational and career guidance policies in the recovery period.

### 1.5 Research hypotheses

Although some research points to orientation toward emerging fields, other studies highlight declines in aspirations, future projections, and career planning. Recognizing that the available research is hardly conclusive and that opposite expectations could also be proposed, we predict the following changes in dimensions related to career aspirations from values before the pandemic (MERPAS 1: 2018–2019) to values measured during the pandemic (MERPAS 2: 2021 and MERPAS 3: 2022), among eighth graders in Bihor County, Romania:

*H1*: Decline in career certainty—supported by evidence that career uncertainty among adolescents had increased significantly and was projected to be further exacerbated during the period of COVID-19 (Mann et al., 2020; Ashta et al., 2023);

*H2*: Decline in career optimism—as demonstrated by findings that adolescent future optimism declined during the pandemic a trend associated with concerns about career and educational opportunities (von Soest et al., 2022);

*H3*: Decline in occupational aspirations—evidenced by studies showing that students lowered their educational goals post-pandemic and experienced erosion of educational aspirations observed alongside to lockdown challenges (Glick et al., 2025; Barn et al., 2023);

*H4*: Changes in occupational aspirations toward medical professions—as indicated by research documenting increased interest in healthcare careers corresponding with the visibility and societal recognition of such roles during the pandemic (Zhang et al., 2022; Wang et al., 2024b; Hamzah et al., 2022; Baskar et al., 2022).

### 1.6 Theoretical framework and empirical evidence

The evolution of career aspirations among teenage students relates to the demand for education and qualifications, a topic of particular interest to scholars within the human capital tradition. Human capital theory provides a framework for understanding this demand, emphasizing education as an investment decision based on a cost–benefit analysis (Becker, 1964). According to Becker (1964), individuals make educational decisions by weighing the expected returns on their investment in education, such as higher earnings and improved employment prospects, against the direct and indirect costs, including tuition fees and forgone earnings during their studies. Crises, such as financial downturns and pandemics, significantly impact this calculation by altering both expected benefits and perceived costs.

The model proposed by Becker (2009) supports both an increase and a decrease in educational demand, depending on the relative strength of financial constraints, opportunity costs, and perceived returns on education. The COVID-19 pandemic was unique in that it lowered opportunity costs (fewer job opportunities) while also introducing new barriers, such as financial struggles, technology gaps, and psychological stress.

Therefore, human capital theory (Becker, 1964) supports the central concept of our study that the investment in education, training, and experience generates future ‘performance.’ In this framework, foundational career optimism is understood as the belief in one’s ability to complete this educational investment. However, at the same time, the theory acknowledges that these outcomes are uncertain (*career uncertainty*), are not guaranteed, and depend on constantly changing factors (such as social networks, structural privileges, or systemic barriers etc.).

Another theory that brings a relevant perspective on the issue of optimism and uncertainty in careers is the Covid-19 Career Shock Theory (Akkermans et al., 2018; Akkermans et al., 2020), which frames the pandemic as a ‘career shock’ or disruptive external event.

This theory emphasizes that young people’s navigation between optimism (their core belief in a positive future) and uncertainty (the career shock) will depend on their ‘career adaptability’ (Savickas, 1997), the ability to adjust their career strategies in response to new contextual barriers. However, it should be noted that a high level of professional insecurity could exhaust adolescents’ foundational career optimism (Greenhalgh and Rosenblatt, 1984).

### 1.7 Evidence of declining career aspirations

The COVID-19 pandemic has significantly altered the landscape of career aspirations among adolescents, leading to both declines and, in some cases, unexpected improvements in their plans. Hussong et al. (2021) and later Carey et al. (2023) conducted literature reviews drawing from peer-reviewed articles, policy reports, and qualitative and quantitative studies published between 2020 and 2023 (Carey et al., 2023; Hussong et al., 2021). Their reviews highlight that the pandemic not only provoked learning loss and feelings of uncertainty but also led to shifts in both short- and long-term educational and career aspirations. Their findings align with prior longitudinal research, demonstrating that economic and social disruptions altered students’ decision-making processes. However, there are fewer clear conclusions regarding whether teenagers tended to lower their aspirations due to the disruptions caused by COVID-19.

Glick et al. (2025) analyzed longitudinal survey data from children and adolescents in Mexico and Nepal affected by school closures. They found that 34% of students lowered their educational goals post-pandemic, with aspirations for higher education dropping by 18 percentage points among lower-income students. Adolescents primarily adjusted their educational goals due to household economic constraints. Research by Fakkel et al. (2023) partially corroborated these findings. Although they did not find that socioeconomic status (SES) explained changes in adolescents’ future orientations, they observed that adolescents who experienced a steeper decline in parental support showed a notable decrease in their positive future orientation.

The interplay between economic strains and aspirations is further illustrated by the narratives provided by Barn et al. (2023), which highlight how urban middle-class children from India experienced an erosion of their educational aspirations due to the challenges posed by lockdowns. In Norway, von Soest et al. (2022) conducted a nationwide, repeated cross-sectional study using data from the Norwegian Ungdata surveys (2014–2021), covering 227,258 adolescents aged 13–18 across 157 municipalities. Their study found that adolescent future optimism declined during the pandemic due to concerns about career and educational opportunities during an economic downturn. The decline in career optimism does not appear to be a direct effect of the pandemic. Mann et al. (2020), who examined career preparedness using pre-pandemic data from the British Cohort Study (1970) and the Organization for Economic Cooperation and Development, Programme for International Student Assessment (OECD PISA) 2018 survey, found that career uncertainty among adolescents had increased by 81% since 2000, particularly among disadvantaged youth. Their study projected that COVID-19 would further exacerbate this uncertainty. Conversely, other studies have highlighted increased educational uncertainty. Ashta et al. (2023) surveyed 666 high school students in Georgia, U. S., and found that 44% of those from low-income backgrounds (FRL-eligible) experienced a decline in confidence regarding college admissions and career opportunities.

### 1.8 Evidence of maintained or enhanced aspirations

In contrast, some studies suggest that the pandemic catalyzed shifts in career aspirations toward sectors perceived as more stable, particularly healthcare. Wang et al. (2024b) note a significant interest among youth in pursuing careers in healthcare, driven by the visibility and importance of such roles during the pandemic, emphasizing the growth of healthcare career aspirations among adolescents. This phenomenon was echoed by Hamzah et al. (2022) who explored the impact of the pandemic on self-efficacy and career aspirations during crises. The heightened awareness of public health challenges during COVID-19 prompted many adolescents to reconsider their career paths, potentially leading to an increase in aspirations in health-related fields. Henkens et al. (2022) reported that Dutch adolescents exhibited a “two-track thinking” approach, acknowledging economic and social disruptions while remaining optimistic about their personal futures. This optimism allowed them to maintain their educational ambitions despite short-term challenges. Additionally, Desrochers et al. (2020) found that Science, Technology, Engineering, and Mathematics (STEM) students in the U.S. largely retained career optimism, although they expressed concerns about the quality of online learning and the loss of hands-on training. O'Malley et al. (2021) found that young adults in Canada, despite facing financial uncertainty and online learning challenges, remained largely optimistic about their future educational and career aspirations. Qureshi et al. (2021) conducted a qualitative study providing a comparative analysis of public and private school students in Pakistan, demonstrating that socio-economic background played a crucial role in shaping career aspirations. Similarly, Cherian and Kohli (2022) examined adolescents from lower socio-economic backgrounds in Bengaluru, India, finding that respondents preferred low-skill jobs, such as shop assistants and mechanics, over careers that required more extensive education.

Not all studies specify the direction of change in career plans, although they record significant shifts in aspirations. Akbari and Faisal (2023), based on a survey of 1,036 Indonesian young people aged 16–30, found that while participants faced heightened anxiety about their future, 87% maintained their pre-pandemic career goals, albeit with a newfound interest in digital entrepreneurship and public health careers. In the UK, Yarde et al. (2022) surveyed 12,828 students and found that nearly 60% altered their career aspirations due to the pandemic, with disadvantaged students facing the most significant disruptions. Similarly, Zhang et al. (2022) conducted a large-scale survey of 21,085 Chinese high school students and found a surge in interest in medical careers, attributed to societal recognition of healthcare professionals during the pandemic. Baskar et al. (2022) surveyed 859 secondary school students in the UK and found a notable shift toward healthcare professions, particularly among female students, due to increased societal recognition and job security in the medical field. Fehkührer et al. (2023) examined how the COVID-19 pandemic shaped adolescents’ concerns and hopes for the future in a survey of 3,052 adolescents in Austria. They found that 54% expressed hope regarding their careers, while 37.7% emphasized that they wanted to be financially secure (Fehkührer et al., 2023).

## 2 Methods

### 2.1 Type of study, design, and approach

This research employs a longitudinal, observational, quantitative study design utilizing data from three waves of the MERPAS survey. The longitudinal approach enables the examination of changes in adolescents’ educational and career aspirations over time by comparing results across multiple data collection points. The study is observational in nature, as it measures naturally occurring phenomena without experimental manipulation, focusing on tracking the same population of 8th-grade students (ages 13–15) in Bihor County across different time periods. The quantitative methodology involves the systematic collection and statistical analysis of numerical data, including the use of standardized occupational status measures (ISEI and SIOPS scales) as indicators of desired occupations. The temporal design is particularly significant as it captures pre-pandemic aspirations (December 2018–January 2019) and compares them with aspirations during peak COVID-19 restrictions in Romania (May–June 2021 and January–February 2022), allowing for analysis of how external societal disruptions may influence adolescent career development trajectories.

### 2.2 Object of study

The unit of analysis in this research is individual adolescent students participating in the MERPAS surveys. The study focuses on analyzing data collected directly from student participants regarding their educational and career aspirations, with each student serving as a distinct case for analysis.

The main objective of this study is to examine the evolving landscape of career aspirations among Romanian eighth-grade students by analyzing survey data collected across three distinct time points: before and during the COVID-19 pandemic, through a longitudinal approach, as we see in Table 1.

**Table 1:** MERPAS data: sample sizes and weighting across multiple series (2018–2022).

MERPAS	Data collection	Size of sample in the current research	Weighted size of sample in current research
MERPAS 1	December 2018–January 2019	1,647	1,643
MERPAS 2	May–June 2021	928	922
MERPAS 3	January–February 2022	1,220	1,217

As specific objectives, we set out to assess whether during the pandemic career uncertainty increased and career optimism (understood as foundational educational commitment) and career strategies (understood as mobility intentions) among Romanian adolescents in Bihor County were altered., to identify changes in the *perception of the status of desired occupations*, and to examine changes in *students’ intentions to study or work abroad*.

### 2.3 Participants

With regard to the participants used in this study, we took into account several factors that could significantly influence the educational and professional aspirations of adolescents and whose significant variation across the three cohorts could alter the comparability of results and would require weighting of the samples. These factors include the gender of the participants, their average age, the type of school they attend, and the locality where the parents of the participating adolescents live. Each factor is represented in the three MERPAS samples (see Table 2), as detailed below.

- Gender: The percentages of female and male participants are almost equal in all cases, with slight variations. MERPAS 1 and 2 show a slightly higher representation of women, while MERPAS 3 is evenly divided, with 50.0% for each gender. Thus, the sample is balanced in terms of gender distribution.
- Average age: The average age of participants varies slightly between samples, with MERPAS 1 having an average age of 14.01 years, MERPAS 2 of 14.39 years, and MERPAS 3 of 14.22 years.
- Type of school: The distribution of participants by type of school indicates that the largest proportion of students comes from secondary schools (gymnasium), consistently exceeding 38% in all three MERPAS groups. National colleges also have a significant representation, with values fluctuating around 22–23%. In contrast, technical colleges have the lowest participation, ranging from 4.4 to 4.6%. Other types of schools— such as technological, theoretical, and theological/vocational high schools—show relatively stable percentages, with minor variations. Overall, the data suggests a predominance of students from general education institutions over specialized ones, with a relatively homogeneous distribution across the three MERPAS samples. One should not confound the type of schools with track or specialization: all data were collected from students in the eighth grade, the last year of the lower secondary cycle in Romania, who can be enrolled in various types of schools classified according to the tracks in the upper secondary.
- Residence: Most parents live in villages or communities, with MERPAS 1 recording the highest percentage (55.2%), followed by MERPAS 3 (54.8%) and MERPAS 2 (53.4%). Small towns have the lowest representation, with percentages ranging from 14.5% (MERPAS 1) and 14.7% (MERPAS 3) to 15.5% (MERPAS 2). Parents living in large cities, such as Oradea, represent approximately 30%, with MERPAS 2 recording the highest proportion (31.1%). Overall, the data reveals a similar distribution in terms of the type of locality in which the parents of the study participants live in the three MERPAS samples, with minor differences.

**Table 2:** MERPAS data: sample structure multiple series (2018–2022).

Sample structure		MERPAS 1%	MERPAS 2%	MERPAS 3%
Gender	Male	49.5	49.6	50.0
	Female	50.5	50.4	50.0
Average age		14.01	14.39	14.22
School Type	National college	22.2	20.9	23.4
	Technical college	4.5	4.6	4.4
	Technological high school	7.5	7.9	7.2
	Theoretical high school	10.8	10.5	10.4
	Vocational high school, Theological	16	15.9	16.6
	Secondary school	39.1	40.2	38.1
Residence	Village/commune	55.2	53.4	54.8
	Small town	14.5	15.5	14.7
	Oradea or another big city	30.3	31.1	30.5

The data were collected using a stratified random sampling approach based on the type of locality and the type of educational institution. For the current longitudinal approach, we have used a pooled database, in which the three subsamples corresponding to the three waves were weighted according to gender, type of residence (rural, small urban, and Oradea/large urban), and type of school (gymnasium school, theoretical high school, technological high school, national college, and technical college) to ensure maximum similarity between waves having wave 2 as reference for the other two surveys. Understandably, the rationale in handling the samples was not representativity but similarity and ceteris paribus comparability across surveys. Wave 2 has been used as reference in a rather arbitrary way, though we considered also the fact that it was closest to the population structure considering the weighting variables. Standard errors of mean for focus variables were only marginally affected by weighting (i.e., ISEI professional aspirations; Table 3).

**Table 3:** S.E of mean: weighted/unweighted variables.

Dataset	S.E. of ISEI of desired profession (unweighted)	S.E. of ISEI of desired profession (weighted)
Merpas 1	0,714	0,709
Merpas 2	0,895	0,903
Merpas 3	0,449	0,449

The MERPAS study collected data from adolescent participants across three waves: MERPAS 1 (December 2018–January 2019, *n* = 1,647, weighted *n* = 1,643), MERPAS 2 (May–June 2021, *n* = 928, weighted *n* = 922), and MERPAS 3 (January–February 2022, *n* = 1,220, weighted *n* = 1,217). The sample maintained balanced gender representation across all waves, with MERPAS 1 and 2 showing slightly higher female participation, while MERPAS 3 achieved perfect gender parity (50.0% each).

### 2.4 Instruments

The primary data collection instrument was the MERPAS survey questionnaire, designed to gather comprehensive information on adolescents’ educational and career aspirations, dropout expectations, migration intentions, and occupational preferences. The questionnaire administration evolved across the three waves to maintain consistency and quality control:

- In MERPAS 1, data collection utilized a mixed approach combining supervised paper-based questionnaires with online self-administration via Google Forms, both conducted under teacher supervision during regular school hours.
- For the subsequent two waves (MERPAS 2 and 3), the data were gathered exclusively through online data collection forms, maintaining the supervised school-based administration format.

This instrument serves as the foundation for longitudinal comparison of career aspirations and educational expectations, enabling us to assess changes in adolescent career development across the three time periods.

To measure occupational aspirations quantitatively, we employed two established occupational status measurement scales. The International Socio-Economic Index of Occupational Status (ISEI) and the Standard International Occupational Prestige Scale (SIOPS) were utilized following the methodology outlined by Ganzeboom et al. (1992). The ISEI measures the socioeconomic status and prestige of desired occupations, while SIOPS provides an alternative measure of occupational prestige. Together, these instruments offer complementary assessments of occupational status aspirations, allowing for robust cross-temporal comparisons and validation of findings regarding changes in the prestige level of students’ career goals/aspirations.

### 2.5 Procedure

Conducted in collaboration with the Bihor County School Inspectorate and the Bihor County Center for Educational Resources and Assistance, the MERPAS surveys are Omnibus research endeavors in which these three institutions engage in a close and continuous partnership throughout the entire research process. To date, three surveys have been deployed, which are used in this article, with samples of various sizes extracted from the population of 8th graders in Bihor County (see Table 1).

With regard to measuring career uncertainty through “I do not know” responses for the desired profession, this approach (proxy) is broadly applied in the research in the field, even in OECD-led surveys (Readiness and OECD, 2024) being also used by many other researchers (Santos et al., 2014; Xu and Tracey, 2015; Fabio et al., 2013; Xu, 2020), one of the best known being Osipow et al. (1976), who developed the Career Decision Scale (CDS), which includes items on uncertainty through responses such as “I do not know what career I want” or “I am confused about my career options.”

Given the sociological, cohort-level design of this study, we adapt the concept of Career Optimism from a purely psychological trait (e.g., Eva et al., 2020) to a sociological-level assessment of viable future paths based on the social context. We operationalize this concept in two distinct components:

1. Core Foundational Optimism: This is the positive assessment of one’s ability to successfully complete the necessary intermediate studies. For 8th graders, any future career requires completing education. Therefore, we use the ‘prospect of school dropout’ as a proxy for this foundational optimism. A low expectation of dropout (Eicher et al., 2014) is interpreted as a resilient positive projection at the cohort level.
2. Career Adaptability / Strategic Planning: This refers to the evaluation of the optimal career path (e.g., domestic vs. international) in response to a changing social context. The pandemic is framed as a ‘career shock’ (Akkermans et al., 2020). We use ‘prospects of migration for education’ and ‘prospects of migration for work’ not as direct measures of optimism, but as indicators of career strategy. A shift in these intentions is hypothesized to reflect a rational ‘career adaptation’ (Savickas, 1997) to new structural barriers (e.g., health risks, travel restrictions), rather than a decline in optimism.

As already stated, occupational aspirations were measured through the ISEI (International Socio-Economic Index of Occupational Status) and SIOPS (Standard International Occupational Prestige Scale) measures of the profession desired at 30.

The International Socio-Economic Index of Occupational Status (ISEI) and the Standard International Occupational Prestige Scale (SIOPS) are both standardized, internationally comparable sociological metrics for assessing occupational status, developed by the same core researchers (Ganzeboom et al., 1992). However, they differ fundamentally in the specific dimension of status they measure and the methodology used to derive their scores. The primary difference between them lies in the criteria used to score the occupations. However, the two indices are very highly correlated. The scales were notably renewed by their creators in 1996 for compatibility with the International Standard Classification of Occupations (ISCO-88).

Two coders independently coded the open-ended occupational aspiration responses into ISCO categories. Inter-coder reliability was assessed separately for each dataset. Agreement between coders was consistently high, ranging from 92.86 to 97.10%. When weighted by the number of cases in each dataset, the overall agreement reached 95.93%, indicating a very high level of consistency in the coding process (see Table 4).

**Table 4:** Inter-coder agreement for VIIT2 responses across MERPAS sub-datasets.

Dataset	VIIT2. [Specify the profession you would like to pursue (or write *I do not know* if applicable)]	*N* (Cases)
MERPAS 1	95.94%	4,708
MERPAS 2	92.86%	1,135
MERPAS 3	97.10%	2,959
Pooled weighted dataset	95.93%	8,802

The analysis of inter-coder agreement across the datasets indicates a consistently high level of concordance between the two independent coders. Both the simple and weighted means exceed 94%, demonstrating robust reliability of the coding process and suggesting that the categorization of items is highly consistent across cases, with larger datasets appropriately influencing the weighted estimates.

Disagreements were resolved through discussion to reach consensus, and the final consensus codes were used for analysis.

The variables used in this study, along with their descriptions and measurements, are presented in Table 5. These include (1) the proxies for career optimism: the *prospect of dropout* which measures expectations of leaving education before obtaining a degree; the *prospect of educational migration* and *work migration,* assessing the likelihood of studying or working abroad; (2) *occupational career uncertainty*, measured by non-responses or ‘Do not know’ answers regarding the desired profession; and (3) *occupational career aspirations*, classified by major International Standard Classification of Occupations (ISCO) groups, level of qualification, and specific medical professions. Finally, the *status of the occupation aspired* is measured using occupational prestige scales (ISEI, SIOPS).

**Table 5:** Indicators of educational and occupational aspirations.

Dimension	Variable	Description, measurement
Career Optimism and Adaptability	Prospect of dropout	Expectation of dropout before obtaining a degree (dichotomy: likely, very likely vs. unlikely, very unlikely)
	Prospect of educational migration	Expectation of studying abroad (dichotomy: likely, very likely vs. unlikely, very unlikely)
	Prospect of work migration	Expectation of working abroad (dichotomy: likely, very likely vs. unlikely, very unlikely)
Career uncertainty	Occupational career uncertainty	Number of “Do not know” and non-responses to the question: Specify the profession you would like to practice (dichotomy: missing vs. non-missing)
Occupational aspirations	Occupational career aspirations—major ISCO group	Desired profession classified in major occupational groups according to ISCO [groups from 0 to 9 according to the International Labor Organization (ILO)]
	Occupational career aspiration—level of qualification	Desired profession classified according to the level of qualification (professions requiring tertiary degrees, secondary level qualifications, other)
	Occupational career aspiration—medical	Desired medical professions (medical professions requiring tertiary degrees, medical professions with lower degrees, other professions)
Status of Occupation aspired	Status of Occupation aspired	Desired profession measured according to occupational prestige scales [numeric, ISEI, SIOPS scales attached to ISCO codes according to Ganzeboom et al. (1992)]. A description of the coding of open-ended questions regarding desired occupation into ISEI and SIOPS scores can be found in Morar (2024).

Based on the measurement of the dependent variables, we propose the following operational hypotheses regarding changes from MERPAS 1 to MERPAS 2 and/or 3:

- Increase in the percentage of missing values for responses to the question regarding desired profession (H1.1)
- Increase in the percentage of those expecting to drop out before acquiring a diploma (testing for a decline in core optimism) (H2.1)
- Increase in the percentage of those expecting to go abroad for work (testing for career adaptation in response to structural barriers) (H2.2)
- Decrease in the percentage of those expecting to go abroad for studies (testing for career adaptation in response to structural barriers) (H2.3)
- Decrease in the average ISEI and SIOPS of the desired profession (H3.1)
- Decrease in the percentage of those desiring professions that require tertiary education (H3.2)
- Increase in the percentage of those opting for medical professions (H4)

### 2.6 Data analysis

The data were collected using a stratified random sampling approach based on the type of locality and the type of educational institution. For the current longitudinal approach, we have used a pooled database, in which the three subsamples corresponding to the three waves were weighted according to gender, type of residence (rural, small urban, and Oradea/large urban), and type of school (gymnasium school, theoretical high school, technological high school, national college, and technical college) to ensure maximum similarity between waves having wave 2 as reference for the other two surveys. Understandably, the rationale in handling the samples was not representativity but similarity and ceteris paribus comparability across surveys.

All data analyses and hypothesis testing were performed using SPSS (Version 25), with the statistical techniques applied to each hypothesis summarized in Table 6.

**Table 6:** Overview of hypotheses tested and corresponding statistical approaches.

Hypotheses	Tests used	Method
H 1.1	Chi-square test	To compare the proportions of valid responses and missing values (e.g., “Do not know” or “No answer”) for the desired profession question across MERPAS 1, 2, and 3.
H 2.1		To compare the percentages of students expecting to drop out across all three survey waves.
H 2.2		To compare the proportion of students expressing a likelihood of seeking employment abroad across the three waves.
H 2.3		To compare the proportion of students considering studying abroad across the three waves.
H 3.1	ANOVA	To compare the average ISEI and SIOPS scores of desired professions across the three survey waves
H 3.2	Chi-square test	To examine the distribution of professional aspirations by the required level of qualification (tertiary education, secondary education, etc.) across the three waves.
H 4		To analyze the percentage of students aspiring to medical professions requiring higher education across the three waves.

### 2.7 Ethical considerations

Ethical approval was obtained from the Doctoral School of Sociology (University of Oradea), the Bihor County School Inspectorate, and the Bihor County Center for Educational Resources and Assistance (no approval number issued); no additional permissions were required. Informed consent was obtained at the time of questionnaire completion, with participants contacted in Bihor County schools to complete the questionnaires online or on paper under supervision.

## 3 Results

We will present the results of the current investigation, beginning with the univariate descriptives of the research variables (see Table 7), followed by the bivariate tests of the hypotheses.

**Table 7:** Univariate distributions of variables in the study.

Variable	Categories	*N*	*N* %
MERPAS survey	MERPAS 1	1,643	43.4
	MERPAS 2	922	24.4
	MERPAS 3	1,217	32.2
Prospect of work migration abroad	Very Unlikely, Unlikely	1762	53
	Likely, Very Likely	1,561	47
Prospect of educational migration abroad	Very Unlikely, Unlikely	2,191	67.6
	Likely, Very Likely	1,050	32.4
Prospect of dropout	Very Unlikely, Unlikely	3,232	95
	Likely, Very Likely	171	5
Aspirations uncertainty—what profession do you desire to practice	Valid	2,400	63.5
	Missing	1,382	36.5
Occupational career aspirations—major ISCO group	Armed forces	63	1.7
	Members of the legislature, the executive, senior public administration officials, senior officials, and civil servants	98	2.6
	Specialists in various fields of activity	1,240	32.8
	Technicians and other technical specialists	216	5.7
	Administrative officers	14	0.4
	Service sector workers	494	13.1
	Skilled agricultural, forestry, and fishery workers	30	0.8
	Skilled and related workers	195	5.2
	Plant and machine operators, machine and equipment assemblers	48	1.3
	Elementary occupations	2	0.10.1
	Missing	1,382	36.5
Occupational career aspiration—level of qualification	Professions with higher education	1,376	36.4
	Professions with secondary education	1,021	27
	Professions without qualifications	2	0.1
	Do not know/Do not answer	1,382	36.5
Occupational career aspiration—medical	Healthcare professions	320	8.5
	Other healthcare professions	19	0.5
	Other	2061	54.5
	Do not know/Do not answer	1,382	36.5
Status of Occupation aspired		Average	Std.dev
	ISEI	61,63	22.04
	SIOPS	52,5	14.64

### 3.1 Univariate results: distributions of study variables

Nearly half of the interviewed students considered working abroad, while a third contemplated studying abroad. However, approximately 5% of the pooled sample indicated they were likely to drop out of school before obtaining a degree.

Furthermore, over a third of the pooled sample did not provide a valid answer when asked about their desired profession, indicating career uncertainty.

Additionally, more than a third of the 8th graders expressed a desire to work in occupations that require a higher education degree. Among this group, 36.5% aimed for medical professions, with nearly one quarter (8.5%) specifically targeting healthcare roles.

In terms of occupational status, the desired occupation had an average ISEI value of 61.3 and an average SIOPS of 52.5, corresponding to mid-level managers or administrators, experienced secondary-level teachers, technical specialists or associate professionals, and certain health professionals such as registered nurses (Ganzeboom et al., 1992).

### 3.2 Bivariate results: comparison across surveys

The operational hypotheses of our research test the prediction that, following the economic and social disturbances corresponding with the COVID-19 pandemic, all indicators of career aspirations have declined from the pre-COVID-19 survey (MERPAS 1) in the subsequent two surveys (MERPAS 2–2020, MERPAS 3–2022). These hypotheses are being tested in the following bivariate analyses.

*H1.1*: Increase in the percentage of missing values for responses to the question regarding desired profession.

Contrary to expectations, the rate of missing values (Do not know and No answer) did not increase during the pandemic, remaining relatively constant from 39.32% in MERPAS 1 to 39.05% in MERPAS 2 (see Table 8). Moreover, in the last survey, conducted between January and February 2022, the percentage of valid answers increased by 8% (from about 61% in MERPAS 1 and MERPAS 2 to 69% in MERPAS 3). These results do not support our hypothesis; therefore, the hypothesis is rejected (H1.1).

**Table 8:** Aspirations’ uncertainty: Proportions of valid answers and missing values for the question ‘Specify the profession you would like to practice’.

Dataset	Percent valid	Percent missing values (DK and NR)
MERPAS 1	60.68	39.32
MERPAS 2	60.95	39.05
MERPAS 3	69.02	30.98

*p* (Chi-square) > 0.05.

*H2.1*: Increase in the percentage of those expecting to drop out before acquiring a diploma

The percentage of students considering dropping out of school before obtaining a degree does not change significantly across the surveys according to both items tested, as shown in Tables 9, 10. Specifically, the percentage of students who view dropping out of school before obtaining a diploma as unlikely or very unlikely remains high, at 94.6% in MERPAS 1, increasing slightly (but statistically insignificantly) to 95.2% in MERPAS 2 and 95.3% in MERPAS 3.

**Table 9:** Have you considered dropping out of school in the last year?

Dataset	Yes	No
MERPAS 1	6.7	93.3
MERPAS 2	4.84.8	95.2
MERPAS 3	5.1	94.9

*p* (Chi-square) > 0.05.

**Table 10:** In the future, do you think any of the following events are likely to happen to you? [dropping out of school before getting a degree].

Dataset	Very Unlikely, Unlikely	Likely, Very Likely
MERPAS 1	94.6	5.4
MERPAS 2	95.2	4.8
MERPAS 3	95.3	4.7

*p* (Chi-square) > 0.05.

When asked a different question, we have got similar answers: students who consider dropping out (to a probable or very probable extent) are at 5.4% in MERPAS 1, which decreases (statistically insignificantly) to 4.8% in MERPAS 2 and 4.7% in MERPAS 3 (see Table 10). Therefore, our results do not support the hypothesis that the percentage of students expecting to drop out before obtaining a degree will increase (H2.1)—hypothesis rejected.

*H2.2*: Increase in the percentage of those expecting to go abroad for work

The data reveals a significant decline in the proportion of students considering working abroad during the pandemic (see Table 11). Before the pandemic (MERPAS 1), the majority (56.6%) expressed a likelihood of seeking employment abroad. However, this figure dropped sharply in MERPAS 2 (38.5%) and remained low in MERPAS 3 (40.8%). The chi-square test confirms that these shifts are statistically significant (*p* < 0.01), supporting the interpretation that students’ willingness to pursue work opportunities abroad was reduced during the pandemic. Thus, our hypothesis that the pandemic increased the percentage expecting to work abroad is rejected: Hypothesis rejected (H2.2).

**Table 11:** In the future, do you think any of the following events are likely to happen to you? [Going to work abroad.]

Dataset	Very Unlikely, Unlikely	Likely, Very Likely
MERPAS 1	43.4	56.6
MERPAS 2	61.5	38.5
MERPAS 3	59.2	40,8

*p* (Chi-square) < 0.01.

*H2.3*: Decrease in the percentage of those expecting to go abroad for studies

Consistent with the observed decline in students’ willingness to work abroad, a similar trend is evident in the proportion of students contemplating studying abroad. The data indicates a significant decrease in the proportion of students considering studying abroad during the pandemic (see Table 12). In the pre-pandemic period (MERPAS 1), 39.1% of students viewed studying abroad as a likely or very likely option. However, this percentage dropped sharply in MERPAS 2 (27.0%) and remained low in MERPAS 3 (28.0%). The chi-square test (*p* < 0.01) confirms that these changes are statistically significant.

**Table 12:** In the future, do you think any of the following events are likely to happen to you? [Going to study abroad.]

Dataset	Very Unlikely, Unlikely	Likely, Very Likely
MERPAS 1	60.9	39.1
MERPAS 2	73.0	27.0
MERPAS 3	72.0	28.0

*p* (Chi-square) < 0.01.

Despite the global emphasis on remote learning and increased accessibility to international education opportunities, these results suggest that students’ concerns about studying abroad may have been heightened during the pandemic. Travel restrictions, financial uncertainties, and general instability could have contributed to this shift. However, since the decline is not as steep as in the case of working abroad, long-term aspirations for international education may remain intact, even if short-term feasibility concerns have influenced students’ immediate expectations.

Given these findings, the initial hypothesis predicting a strong decrease in study-abroad intentions is confirmed: Hypothesis confirmed (H2.3).

*H3.1*: Decrease in the average ISEI and SIOPS of the desired profession

**Figure 1:**
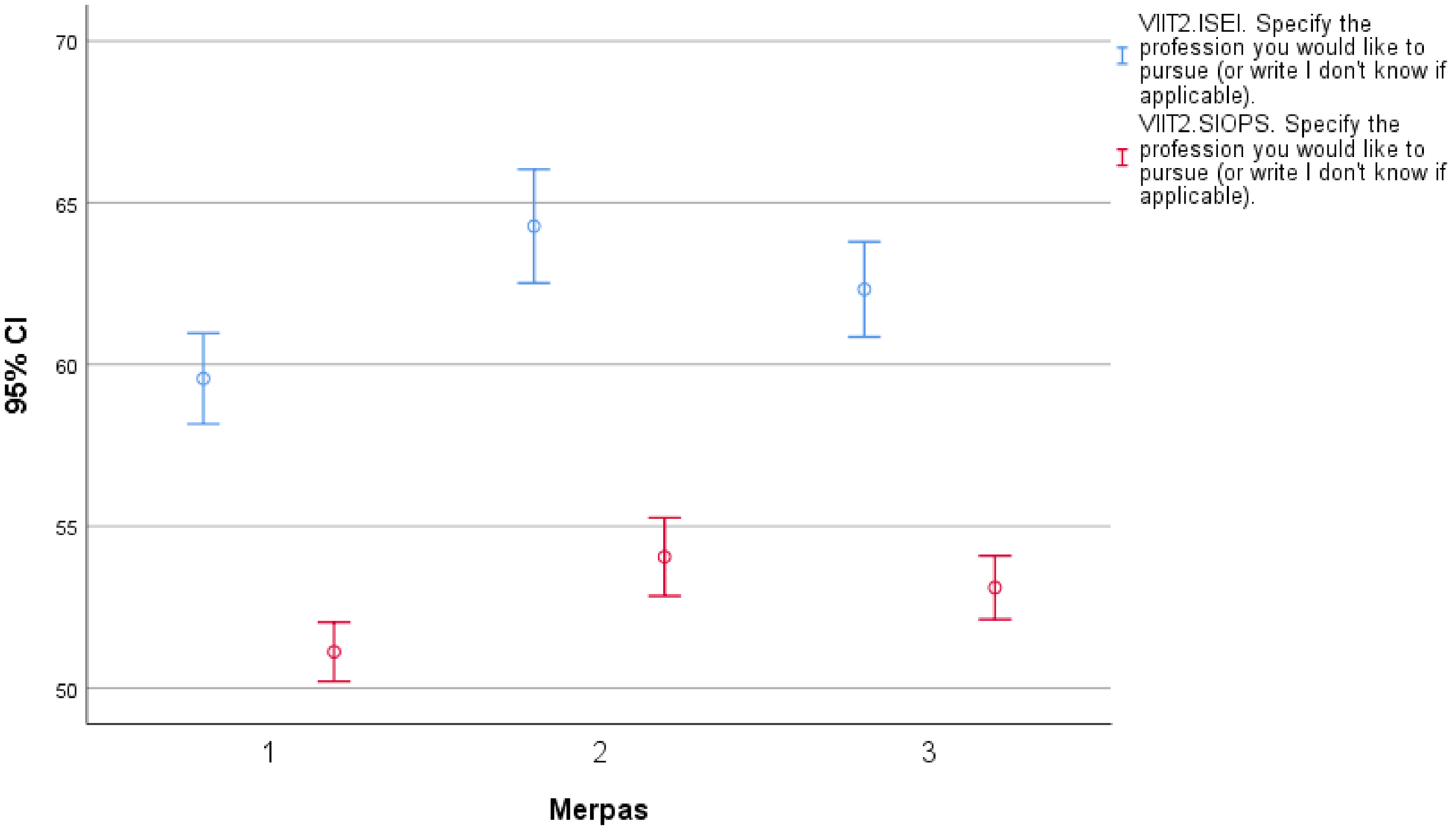
Average ISEI and SIOPS scores of the desired profession.

Aligned with some of the theoretical expectations, the status of the desired occupation significantly increased during the pandemic. MERPAS 2 shows the highest scores in both measures, and the ANOVA results indicate significant differences between the groups (*p* < 0.01) (see Table 13; Figure 1). These findings suggest that there are meaningful variations in occupational preferences or expectations among the groups, implying that professional aspirations differ significantly across the groups: Hypothesis rejected (H3.1).

**Table 13:** Average of ISEI/SIOPS score of desired occupation.

Dataset	Specify the profession you would like to practice (ISEI score)	Specify the profession you would like to practice (SIOPS score)
MERPAS 1	59.56	51.12
MERPAS 2	64.27	54.05
MERPAS 3	62.32	53.10

*p* (Chi-square) < 0.01.

*H3.2*: Decrease in the percentage of those desiring professions that require tertiary education

The distributions of professional aspirations remain remarkably stable across the three surveys, with little significant change, summarized as a small increase in tertiary-educated professions in MERPAS 2 (during the pandemic) and a decline in options for professions requiring secondary-level education. This is more evident in Table 14. Therefore, during the pandemic, occupational aspirations did not decline; on the contrary, they seem to have increased and become clearer. Hypothesis rejected (H3.2).

**Table 14:** Professional aspirations (ISCO major categories) by survey (%).

1	MERPAS 1	MERPAS 2	MERPAS 3
Armed Forces	2.1	3.0	3.0
Members of the legislature, the executive, senior public administration officials, senior officials, and civil servants	2.4	4.4	5.9
Specialists in various fields of activity	51.1	55.2	50.1
Technicians and other technical specialists	9.5	7.4	9.4
Administrative officers	0.6	0.8	0.4
Service sector workers	22.2	19.1	19.7
Skilled agricultural and fishery workers	1.2	1.4	1.2
Skilled and related workers	8.2	6.8	8.9
Plant and machine operators, machine and equipment assemblers	2.7	1.6	1.5
Elementary occupations	0.1	0.3	0.0

*p* (Chi-square) < 0.05.

In MERPAS 1, 33.4% preferred higher education, 27.3% sought secondary education, 0.1% wanted jobs with no qualifications, and 39.3% were unsure or did not respond. In MERPAS 2, 37.9% preferred higher education, 23% preferred secondary education, 0.2% were interested in unqualified jobs, and 38.9% did not answer. In MERPAS 3, 39.3% wanted higher education, 29.7% sought secondary education, 0% preferred unqualified jobs, and 31.1% were unsure. The Chi-square test result (p < 0.01) indicates statistically significant differences in preferences among the groups, as shown in Table 15.

**Table 15:** Level of qualification of desired job by survey (%).

Dataset	Professions with higher education	Professions with secondary education	Professions without qualifications	Do not know/Do not answer
MERPAS 1	33.4	27.3	0.1	39.3
MERPAS 2	37.9	23.0	0.2	38.9
MERPAS 3	39.3	29.7	0.0	31.1

*p* (Chi-square) < 0.01.

*H4*: Increase in the percentage of those opting for medical professions

Regarding aspirations for medical professions, the results show a slight increase in the percentage of students aspiring to the category of these professions requiring higher education across the three survey waves, from 7.6% (MERPAS 1) to 9.2% (MERPAS 3), as shown in Table 16. In contrast, the percentage of respondents considering other healthcare professions remained very low, with values of 0.8, 0.2, and 0.4%. Additionally, a significant portion of respondents, ranging from 31.1–39.3%, reported uncertainty or preferred not to answer. The Chi-square test result (p < 0.01) indicates that these differences are statistically significant. Despite the increase in interest in medical professions, the rise is much smaller than the broader trend observed for other professions during the pandemic, as well as the overall increase in career certainty: Hypothesis rejected (H4).

**Table 16:** Aspirations for medical professions by level of qualification and survey (%).

Dataset	Healthcare professions with tertiary qualifications	Other healthcare professions	Other (non-healthcare professions)	Do not know/ Do not answer
MERPAS 1	7.6	0.8	52.4	39.3
MERPAS 2	9.1	0.2	51.7	38.9
MERPAS 3	9.2	0.4	59.4	31.1

*p* (Chi-square) < 0.01.

## 4 Discussion

The objective of this study was to examine changes in adolescents’ career aspirations, occupational status perceptions, and international mobility intentions by comparing pre-pandemic and during-pandemic data. In order for this, four general hypotheses were formulated and subsequently operationalized into specific hypotheses aligned with the dependent variables. Thus, the present discussion addresses the results of the tested hypotheses in light of the literature review.

### 4.1 H 1: decline in career certainty

*H 1.1*: Increase in the percentage of missing values for responses to the question regarding desired profession—rejected.

Contrary to initial expectations and numerous international studies, this research found an improvement in students’ clarity regarding their career goals during the pandemic. Specifically, the rate of missing values for desired professions—measure proxy utilized for uncertainty—did not increase; rather, it remained relatively constant between MERPAS 1 (pre-pandemic) and MERPAS 2 (during height of restrictions), and then significantly decreased from 39.32 to 30.98% in MERPAS 3 (just before restrictions ended), as seen in Table 8. This suggests a higher proportion of students were able to provide valid responses about their desired professions as the pandemic progressed.

This finding directly contradicts evidence from several international studies, which suggested that the COVID-19 pandemic would lead to increased career uncertainty among adolescents. For instance, Mann et al. (2020) projected that the pandemic would exacerbate existing career uncertainty. Similarly, Carey et al. (2023) highlighted that the pandemic provoked feelings of uncertainty and altered adolescents’ short- and long-term plans. The Romanian context appears to have fostered greater clarity, standing apart from these broader trends.

### 4.2 H 2: Decline in career optimism

*H 2.1*: Increase in the percentage of those expecting to drop out before acquiring a diploma—rejected.

Regarding career optimism, measured by the intention to drop out of school, our study found that the percentage of students considering dropping out before obtaining a degree did not change significantly across the three survey waves, consistently remaining low (around 5%), as we can see in Tables 9, 10. This indicates a rejection of the hypothesis that the pandemic would increase the likelihood of students dropping out before acquiring a diploma.

This stability in dropout intentions contrasts with international findings that reported declining future optimism among adolescents due to concerns about career and educational opportunities during economic downturns (von Soest et al., 2022). The resilience observed in Romanian adolescents’ foundational optimism is theorized to be linked to several factors:

- Economic resilience: The economic factor may be decisive, particularly the limited impact of the pandemic and the relatively rapid recovery of the economy. Romania’s GDP contraction was smaller than in other European countries in 2020, and the subsequent recovery was stronger (Eurostat, 2024; Negrea and Bekesi, 2020; Siegel, 2024; Union, E, 2024). This aspect could have supported *social optimism in general*.
- Parental job stability: Additionally, adolescents’ observations of their parents adapting to remote work and maintaining job stability may have mitigated the negative impact on career confidence (Wang et al., 2024a; Liang et al., 2020). Since our study is a longitudinal trend study and not a panel study, we cannot investigate the differential impact of economic hardship on specific categories of students from different socio-economic backgrounds. It is possible that the effect on career aspirations was much larger for those who suffered most from business closures (Fairlie, 2020; Akkermans et al., 2020; Bartik et al., 2020).
- Cultural and family resilience: The optimism of adolescents in the MERPAS study regarding occupational prospects may be linked to cultural or family resilience mechanisms activated during the pandemic, as suggested by the literature (Hussong et al., 2021; Carey et al., 2023).
- Adaptation to online learning: Furthermore, although Romania experienced prolonged school closures, the data do not show a decrease in aspirations. This can be explained by the rapid adjustment to online learning and the use of virtual technologies, which allowed students to discover new digital skills (Cosma and Hatos, 2022; Lazăr and Roman, 2021). The time spent at home may have meant a more direct family involvement in discussions about school or career, thereby strengthening long-term aspirations—some studies have noted a buffering role for families; see Hussong et al. (2021).

*H 2.2*: Increase in the percentage of those expecting to go abroad for work—rejected.

*H 2.3:* Decrease in the percentage of those expecting to go abroad for studies—confirmed.

Regarding the data indicating a sharp decrease in the proportion of students considering going abroad (for work or studies) we interpret it not as a decline in optimism, but as a clear demonstration of career adaptability. As ‘career shock’ theory (Akkermans et al., 2020) suggests, these adolescents were rationally re-evaluating their career strategies in response to new, acute structural barriers. This trend can be understood as the result of a confluence of psychosocial and structural factors. On a psychosocial level, the global health crisis likely amplified the desire for proximity to family and social support networks, making the prospect of being isolated abroad during a period of high health risk less appealing (Dascalu, 2020; Stănculescu and Marin, 2022). However, this shift in preferences was reinforced by a set of structural barriers: widespread restrictions on international travel (Mantu, 2020) and institutional ambiguity regarding the operational status of universities or foreign employers. Therefore, the observed decline in migration intentions reflects not just a change in aspirations, but a pragmatic response to a global environment where the logistical, financial, and health-related costs of mobility had dramatically increased. These findings suggest that, for the cohorts surveyed in our study, the pandemic period was associated with a notable reduction in career uncertainty and a strengthening of domestic career path commitment. While our trend design does not permit causal claims, the data indicate a shift towards greater clarity and higher-status aspirations occurring concurrently with the pandemic’s profound social and educational disruptions.

This interpretation diverges from some studies, such as Swanson and Duncan (2021), who found that COVID-19 safety issues alone did not diminish interest in study abroad, but rather a broader consideration of risk, financial concerns, and classification, which are impacting future study abroad interest.

### 4.3 H 3: decline in occupational aspirations

*H 3.1:* Decrease in the average ISEI and SIOPS of the desired profession—rejected.

The unexpected increase in aspirations regarding occupational status (ISEI and SIOPS) during the pandemic warrants discussion. The distributions of professional aspirations by required level of qualification remained remarkably stable or even slightly increased for professions demanding higher education, while options for secondary-level education declined (Table 15). Both of these findings led to the rejection of the hypotheses that occupational aspirations would decline.

These findings contradict international studies that observed a lowering of educational goals post-pandemic, especially among lower-income students, due to household economic constraints (Barn et al., 2023; Glick et al., 2025). The observed increase in aspirations for higher-status and higher-qualified professions in Romania is interpreted as a heightened awareness of the importance of higher qualifications or “safe” professions (Baskar et al., 2022; Guszkowska and Dąbrowska-Zimakowska, 2023; Zhang et al., 2022). This aligns with human capital theory (Becker, 1964; Becker, 2009; OCDE, 1998), which suggests that during crises, career aspirations may increase due to the lowering of opportunity costs of education (fewer job opportunities) and increased perceived health risks associated with low- and medium-qualification occupations. This makes safer and better-paid positions (often requiring higher education) more attractive, acting as an investment during uncertain times.

*H 3.2*: Decrease in the percentage of those desiring a profession who acquire tertiary education—rejected.

Contrary to the hypothesis, there was even a small increase in the preference for professions requiring tertiary education during MERPAS 2 (at the height of the pandemic) and MERPAS 3 (later in the pandemic), while options for professions requiring secondary education declined. Specifically, the percentage of students preferring professions with higher education increased from 33.4% in MERPAS 1 (pre-pandemic) to 37.9% in MERPAS 2 and 39.3% in MERPAS 3. These differences were statistically significant (*p* < 0.01), indicating that occupational aspirations for higher education did not decline; instead, they appeared to have increased and become clearer.

This trend aligns with human capital theory (Becker, 1964; Becker, 2009), which supports an increase or stability in occupational aspirations for higher education due to economic resilience and the perceived value of high qualifications. However, international research indicates a decline in optimism and educational goals caused by economic constraints and pandemic uncertainty (Carey et al., 2023; Hussong et al., 2021).

At the same time, the shift to online pedagogy, despite presenting many challenges, has also created new opportunities for tertiary education systems in developing countries. For example, online education has enhanced flexibility for both teachers (in planning and organizing lessons) and for students to learn at their own pace, which can make tertiary education more accessible, inclusive, and resilient (Islam, 2021).

### 4.4 H 4: changes in occupational aspirations toward medical professions

*H 4.1*: Increase in the percentage of those opting for medical professions—rejected.

While some international researchers observed students’ orientation toward medical professions (Baskar et al., 2022; Zhang et al., 2022), the MERPAS data show only a modest increase in the percentage of students aspiring to medical professions requiring higher education. This modest rise, which was much smaller than the broader trend observed for other professions, led to the rejection of the hypothesis (H4). This rejection occurred because the observed increase (from 7.6% in MERPAS 1 to 9.2% in MERPAS 3), despite being statistically significant (*p* < 0.01), was much smaller than the broader trend observed for other professions and the overall increase in career certainty, as we see in Table 16.

Possible explanations include the *stress displayed by the healthcare system* (Savu et al., 2023; Briciu et al., 2023)—overcrowded hospitals and exhaustion were frequently reported in the Romanian media, which may have diminished the attractiveness of medical professions*. Existing perceptions* of medical careers, already *positive* (Savu et al., 2023), remained stable during the pandemic instead of increasing further. Another aspect relates to the *specificity of the analyzed cohort*; these are eighth-grade students, who may view a medical career as requiring intermediate stages (Roșioară et al., 2024; Muntean et al., 2022).

### 4.5 Interpretation

Our interpretation of the evolution of career aspirations among adolescents in Bihor during the pandemic—especially in contrast to trends recorded in international literature—suggests that these developments were predominantly influenced by health concerns rather than negative economic projections. This shift led to a preference for safer professions that require higher education, even if it involved abandoning studies or work abroad. Furthermore, our results support the theories of human capital theory: if the opportunity costs of higher education do not increase while the risks associated with low- and medium-qualification jobs rise, it is logical to opt for safer and better-paid positions, which explains the heightened career aspirations of our respondents.

This serves as a working hypothesis with broad applicability, assuming that the pandemic’s economic impact on the families of our study participants was minimal. Robust testing of this conjecture would necessitate comparisons on at least international cohort data that include measures of the pandemic’s economic impact, as well as population health and safety indicators (such as excess mortality).

A methodological approach to the differences between our findings and the ones found in previous research cannot be overlooked. It is essential to highlight the limitations of earlier studies and the fundamental differences in design and measurement compared to our proposed study. Many of the reviewed studies employed a cross-sectional design, which assessed changes in career aspirations or attitudes retrospectively and/or approached career aspirations from a psychological perspective, focusing on dependent variables such as career optimism, career uncertainty, or proxies of career concern, such as involvement in career counseling and guidance activities. According to theoretical models, contextual factors are crucial—such as the timing of government-imposed restrictions and the severity of the pandemic’s economic and health effects—and their impact could be more effectively tested through international comparative studies.

### 4.6 Limitations

The findings from our study of eighth-grade students in Bihor County present a contrast to many international reports on the pandemic’s impact on youth aspirations. While Bihor County shares many demographic and educational characteristics with other regions in Romania, our results should be interpreted as a regional case study, and caution is warranted against direct generalization to the entire country without further corroborating research. This regional focus provides a valuable, context-specific lens through which to understand how local economic resilience, as noted in Romania’s relatively mild GDP contraction and Bihor County’s similar context may mediate the effects of a global crisis on adolescent career planning.

Also, it is important to interpret these findings with caution regarding causality. Our longitudinal design is a trend analysis (or repeated cross-sectional study), meaning it compares different cohorts of eighth-grade students over time, rather than tracking the same individuals (a panel design). Consequently, we cannot definitively attribute the observed shifts solely to the pandemic’s influence. These changes may also reflect underlying cohort effects, where the unique life experiences and baseline characteristics of the students surveyed in 2021 and 2022 shaped their aspirations differently from the 2019 cohort. Other unmeasured societal factors may also have played a role, therefore, the pandemic should be understood as the dominant historical context (or “career shock”) in which these changes were observed, rather than as a singular causal variable.

Our longitudinal design is a trend analysis, meaning that *the three samples included in the analysis consist of different individuals*, although they are similar in structure. However, it is possible that the dynamics we investigated are influenced by cohort effects rather than changes in individual attitudes, as the theory suggests. This limit has been overcome only by using panel data. The hypothesis that such a great similarity of distributions of occupational aspirations as the ones recorded in our research across three cohorts of 14-year-olds from Bihor County, Romania, would be accidentally produced by the pandemic-induced shifts in aspirations for those questioned in 2022 and 2023, who otherwise have had different aspirations, is very unlikely, though.

The theoretical and methodological limitation of this study is the conceptualization of Career Optimism. We relied on proxy measures (dropout intentions, migration intentions) rather than direct, validated psychometric instruments. While we have reframed the concept of Career Optimism sociologically, as “foundational optimism” and “career adaptation,” this approach presents certain theoretical and methodological issues. This approach does not allow for a differentiation regarding Career Optimism of the cohorts analyzed; it only allows them to establish a positive orientation regarding their career projections. Also, the observed decline in migration intentions is interpreted in our discussion as a response to health insecurity and structural travel barriers, but we cannot fully disentangle this from other factors, such as family financial security, which our study did not measure. Future research, employing panel design and validated psychological scales, would be necessary to isolate these distinct mechanisms that determine Career Optimism in adolescents.

Moreover, one could criticize the generalization of our results, which were based on the analyzes of samples drawn from the student population of Bihor County to the entire similar population of Romania. Although from any point of view the student population of Bihor is highly similar to that of Romania, if we take some indicators like dropout or results at the high-stakes National Evaluation examination, one should be cautious when applying our results to all Romanian students.

Another limitation in the comparability of our study with previous ones is that *we did not directly assess the educational aspirations of our subjects*, although we used the level of qualification of their desired future jobs as a proxy for educational aspirations. However, this issue is non-consequential to our conclusions, as the core of this paper tackles questions regarding occupational aspirations. Even though it is hard to imagine that educational aspirations can be disentangled from occupational desires, therefore, based on the dynamics of occupational aspirations, one could be able to deduce changes in educational aspirations.

Besides these concerns, the specification of career decision and planning focusing only on students’ opinions may limit the scope of our research, as this decision-making process is definitely more of a social sort, involving members of the family and peer group, and many complex social influence processes. However, the rational choice considerations can be applied as well to the results of this process as manifested in the answers of the teenagers, although we have to admit that the aspirations might not be entirely of the adolescent’s but results of their embeddedness into a social group.

## 5 Conclusion

This study examines the evolution of career aspirations among eighth graders in Bihor County—Romania, by comparing pre-pandemic (MERPAS 1) and pandemic-era (MERPAS 2 and MERPAS 3) survey data. Contrary to prevailing international findings that documented a decline in psychological career optimism and increased uncertainty during the COVID-19 pandemic, our results, using a sociological framework, indicate that foundational career optimism (i.e., educational commitment) remained resilient and stable. Furthermore, occupational aspirations for higher-status jobs either remained stable or even improved. Specifically, the clarity of students’ career aspirations increased, as evidenced by a higher proportion of valid responses regarding desired professions, and the average occupational status—as measured by ISEI and SIOPS—showed a slight but statistically significant increase during the pandemic.

Moreover, the data reveal a significant reduction in the inclination among students to pursue educational or professional opportunities abroad. This trend likely reflects the impact of stringent travel restrictions, heightened health concerns, and a reassessment of risk during the crisis. In the Romanian context, these findings may be partially explained by the country’s relatively moderate economic downturn and robust recovery, as well as the rapid adaptation to remote learning modalities and increased family involvement during periods of school closures. These results confirm previous findings that signaled no decline and even heightened aspirations. Theoretical support can also be found in human capital theory, which provides arguments for expectations of improved occupational aspirations during crises, depending on the opportunity costs of education and the relative health risks of specific occupations.

Despite these insights, several limitations of our research must be acknowledged. The use of repeated cross-sectional data, rather than a true longitudinal panel design, may introduce cohort effects that limit the ability to generalize individual-level changes over time. Additionally, the reliance on proxy measures—such as the number of valid responses and required educational qualifications—to gauge career aspirations may not fully capture the multidimensional nature of students’ future orientations. More specifically, as noted in our limitations, our use of “dropout” and “migration” intentions as proxies for Career Optimism and Adaptation, respectively, is a sociological construct that cannot substitute for validated psychological scales.

Future research focusing on how large-scale, societal crises affect processes of career planning should prioritize longitudinal panel studies and incorporate broader international comparisons to further elucidate the complex interplay between economic context, educational disruption, and career aspiration trends during global crises. Our findings underscore the importance of considering local economic and cultural contexts when evaluating the impact of external shocks on youth career trajectories, and that results obtained in other contexts can be applied to local situations only after carefully considering the variations in the relevant variables describing the environment of the dependent variables.

Summing up, our research underlines the complexities of the career planning process in the case of adolescents, highlighting the value of a utilitarian perspective which has, however, to take into account the many detailed stakes that matter to the teenager, and for those around him. Moreover, the fact that Bihor County—Romanian teenagers increased their aspirations during the COVID-19 pandemic reveals the possible role of education, especially of higher education, as a safe-haven investment on behalf of certain categories of youth in uncertain times. These topics deserve further investigation as they could provide valuable insights for training and career counseling activities planning.

## Data Availability

The raw data supporting the conclusions of this article will be made available by the authors, without undue reservation.

## References

[ref1] Akbari T. T. Faisal M. (2023). Shifting career aspirations and anxiety during the Covid-19 pandemic. J. An-Nafs: Kajian Penelitian Psikologi 8, 49–67. doi: 10.33367/psi.v8i1.3022

[ref2] Akkermans J. Richardson J. Kraimer M. L. (2020). The Covid-19 crisis as a career shock: implications for careers and vocational behavior. J. Vocat. Behav. 119. doi: 10.1016/j.jvb.2020.103434, PMID: 32390655 PMC7205633

[ref3] Akkermans J. Seibert S. E. Mol S. T. (2018). Tales of the unexpected: integrating career shocks in the contemporary careers literature. S. Afr. J. Ind. Psychol. 44:1. doi: 10.4102/sajip.v44i0.1503

[ref4] Ashta J. K. Weingart R. Gazmararian J. A. (2023). The impact of COVID-19 on education experiences of high school students in semi-rural Georgia. J. Sch. Health 93, 257–265. doi: 10.1111/josh.13269, PMID: 36414540 PMC10006293

[ref5] Barn R. Sandhu D. Mukherjee U. (2023). Re-imaging everyday routines and educational aspirations under COVID-19 lockdown: narratives of urban middle-class children in Punjab, India. Child. Soc. 37, 254–269. doi: 10.1111/chso.12571, PMID: 35942022 PMC9347931

[ref6] Bartik A. W. Bertrand M. Cullen Z. Glaeser E. L. Luca M. Stanton C. (2020). The impact of COVID-19 on small business outcomes and expectations. Proc. Natl. Acad. Sci. 117, 17656–17666. doi: 10.1073/pnas.2006991117, PMID: 32651281 PMC7395529

[ref7] Baskar A. Quigley L. Sangha K. (2022). The impact of COVID-19 pandemic on career choice in a secondary school wide survey. J. Med. Educ. Res. 2. doi: 10.5750/jmer.v2i1.2042

[ref8] Becker G. S. (1964). Human capital. New York: National Bureau of Economic Research.

[ref9] Becker G. S. (2009). Human capital: A theoretical and empirical analysis, with special reference to education. Chicago, IL: University of Chicago press.

[ref10] Bemme D. Gayer-Anderson C. Irvine A. Strang L. Rose N. (2021). Long-term societal implications of COVID-19: mental health.

[ref11] Briciu V. Leucuta D.-C. Tőkés G. E. Colcear D. (2023). Burnout, depression, and job stress factors in healthcare workers of a Romanian COVID-19 dedicated hospital, after two pandemic years. Int. J. Environ. Res. Public Health 20:4118. doi: 10.3390/ijerph20054118, PMID: 36901130 PMC10001558

[ref12] Carey R. L. Bailey M. J. Polanco C. I. (2023). How the COVID-19 pandemic shaped adolescents’ future orientations: insights from a global scoping review. Curr. Opin. Psychol. 53:101655. doi: 10.1016/j.copsyc.2023.101655, PMID: 37540938

[ref13] Cherian R. M. Kohli Y. (2022). Analysing the shift in aspirations among adolescents during Covid-19 pandemic. CHILDREN FIRST, 55.

[ref14] Chirtoc I. (2021). Mortalitatea în România a crescut cu 30% în septembrie față de perioada prepandemică Ziare.com. Available online at: https://ziare.com/social/coronavirus/mortalitate-romania-covid-pandemie-1710655 (Accessed March 14, 2025).

[ref15] Cockerham D. Lin L. Ndolo S. Schwartz M. (2021). Voices of the students: adolescent well-being and social interactions during the emergent shift to online learning environments. Educ. Inf. Technol. 26, 7523–7541. doi: 10.1007/s10639-021-10601-4, PMID: 34149300 PMC8202218

[ref16] Cosma M.-L. Hatos A. (2022). The Romanian students’ perceptions of digital learning and its consequences on learning efficiency: a qualitative study.

[ref17] Cournoyer D. E. Rohner R. P. (1996). Reliability of retrospective reports of perceived maternal acceptance-rejection in childhood. Psychol. Rep. 78, 147–150. doi: 10.2466/pr0.1996.78.1.147, PMID: 8839309

[ref18] Cristea M. Dănăcică D. E. Noja G. G. (2021). Emigration decision and the migration profile of the unemployed: a case study on Romania. Rom. J. Econ. Forecast. 24:94.

[ref9002] Dascalu S. (2020). The successes and failures of the initial COVID-19 pandemic response in Romania. Frontiers in public health, 8:344. doi: 10.3389/fpubh.2020.00344 32766201 PMC7381272

[ref19] Desrochers M. Naybor D. Kelting D. (2020). Perceived impact of COVID-19 and other factors on STEM students’ career development. J. Res. STEM Educ. 6, 138–157. doi: 10.51355/jstem.2020.91

[ref20] Eicher V. Staerklé C. Clémence A. (2014). I want to quit education: a longitudinal study of stress and optimism as predictors of school dropout intention. J. Adolesc. 37, 1021–1030. doi: 10.1016/j.adolescence.2014.07.007, PMID: 25128662

[ref21] Eurostat (2024). National accounts and GDP. Available online at: https://ec.europa.eu/eurostat/statistics-explained/index.php?title=National_accounts_and_GDP [Accessed February 24, 2025].

[ref9001] Eva N. Newman A. Jiang Z. Brouwer M. (2020). Career optimism: A systematic review and agenda for future research. J. Vocat. Behav. 116:103287. doi: 10.1016/j.jvb.2019.02.011

[ref22] Fabio A. D. Palazzeschi L. Asulin-Peretz L. Gati I. (2013). Career indecision versus indecisiveness: associations with personality traits and emotional intelligence. J. Career Assess. 21, 42–56. doi: 10.1177/1069072712454698

[ref23] Fairlie R. W. (2020). The impact of COVID-19 on small business owners: continued losses and the partial rebound in May 2020.

[ref24] Fakkel M. Peeters M. Branje S. Stevens G. W. Vollebergh W. A. (2023). Decline in positive future orientations among adolescents during COVID-19: the role of socioeconomic status, parental support, and sense of control. J. Adolesc. 95, 1321–1332. doi: 10.1002/jad.12204, PMID: 37321963

[ref25] Fehkührer S. Humer E. Kaltschik S. Pieh C. Probst T. Diestler G. . (2023). Young people and the future: school students’ concerns and hopes for the future after one year of COVID-19 in Austria—findings of a mixed-methods pilot study. Healthcare 11:2242. doi: 10.3390/healthcare11162242, PMID: 37628439 PMC10454506

[ref26] Ganzeboom H. B. De Graaf P. M. Treiman D. J. (1992). A standard international socio-economic index of occupational status. Social science research, 21, 1–56. doi: 10.1016/0049-089X(92)90017-B

[ref27] Gherheș V. Dragomir G.-M. Cernicova-Buca M. (2020). Migration intentions of Romanian engineering students. Sustainability 12:4846. doi: 10.3390/su12124846

[ref28] Glick J. E. Alcaraz M. Randrianasolo A. Yabiku S. T. (2025). COVID-19, school closures and the retreat from educational aspirations. J. Adolesc. 97, 148–164. doi: 10.1002/jad.12406, PMID: 39279281 PMC11701403

[ref29] Greenhalgh L. Rosenblatt Z. (1984). Job insecurity: toward conceptual clarity. Acad. Manag. Rev. 9, 438–448. doi: 10.5465/amr.1984.4279673

[ref30] Guszkowska M. Dąbrowska-Zimakowska A. (2023). Occupational balance, changes in occupations and psychological well-being of university students during the COVID-19 pandemic. Scand. J. Occup. Ther. 30, 463–474. doi: 10.1080/11038128.2022.2143892, PMID: 36354975

[ref31] Hamzah S. R. A. Musa S. N. S. Mohamad N. (2022). The mediating effect of self-efficacy on career aspiration and organizational support with subjective career success among Malaysian women managers during the Covid-19 pandemic. Front. Sociol. 7:802090. doi: 10.3389/fsoc.2022.802090, PMID: 36072501 PMC9442342

[ref32] Hatos A. Gyarmati B. F. (2023). The reliability of self-reported GPA in educational research: a comparison of self-reported and officially recorded data. Rev. Rom. Pentru Educ. Multidim. 15, 159–177. doi: 10.18662/rrem/15.4/786

[ref33] Henkens J. H. Visser K. Finkenauer C. Stevens G. W. (2022). I think it’ll all blow over in the end’: how young people perceive the impact of COVID-19 on their future orientations. Young 30, 309–326. doi: 10.1177/11033088221085137

[ref34] Hussong A. M. Benner A. D. Erdem G. Lansford J. E. Makila L. M. Petrie R. C. . (2021). Adolescence amid a pandemic: short-and long-term implications. J. Res. Adolesc. 31, 820–835. doi: 10.1111/jora.12671, PMID: 34448291 PMC8646616

[ref35] Iacob R. (2018). Brain drain phenomenon in Romania: what comes in line after corruption? Rev. Rom. Comun. Relat. Public. 20, 53–78. doi: 10.21018/rjcpr.2018.2.259

[ref36] International Standard Classification of Occupations (ISCO) (1992). Available online at: https://ilostat.ilo.org/methods/concepts-and-definitions/classification-occupation/ [Accessed March 6 2025].

[ref37] Ion I. E. Lupu R. Nicolae E. (2022). Academic achievement and professional aspirations: between the impacts of family, self-efficacy and school counselling. J. Fam. Stud. 28, 587–610. doi: 10.1080/13229400.2020.1746685

[ref38] Islam S. (2021). Unlearning, relearning, and paradigm shift to online tertiary education during the COVID-19 pandemic in Bangladesh. Bangladesh J. Med. Sci. 65-71, 65–71. doi: 10.3329/bjms.v20i5.55399, PMID: 40208441

[ref39] Kalenkoski C. M. Pabilonia S. W. (2025). Teen social interactions and well-being during the COVID-19 pandemic. Rev. Econ. Househ. 23, 357–404. doi: 10.1007/s11150-024-09712-x

[ref40] Lazăr F. Roman G. V. (2021). Higher education policies for developing digital skills in response to the Covid-19 crisis: the case of Romania. Higher education policies for developing digital skills to respond to the Covid-19 crisis: European and global perspectives, 19–31.

[ref41] Liang Y. Zhou N. Dou K. Cao H. Li J.-B. Wu Q. . (2020). Career-related parental behaviors, adolescents’ consideration of future consequences, and career adaptability: a three-wave longitudinal study. J. Couns. Psychol. 67, 208–221. doi: 10.1037/cou0000413, PMID: 32105127

[ref42] Maftei A. Merlici I.-A. Roca I.-C. (2022). Implications of the COVID-19 pandemic on children and adolescents: cognitive and emotional representations. Children 9:359. doi: 10.3390/children9030359, PMID: 35327734 PMC8946934

[ref43] Mann A. Denis V. Percy C. (2020). Career ready?: how schools can better prepare young people for working life in the era of COVID-19. OECD education working papers OECD Publishing.

[ref9004] Mantu S. (2020). EU citizenship, free movement, and Covid-19 in Romania. Front. Human Dynam. 2:594987. doi: 10.3389/fhumd.2020.594987

[ref44] Mareş G. Cojocariu V.-M. Cîrtiţă-Buzoianu C. (2022). Challenges in the career decision process for teenagers from disadvantaged areas. Rev. Rom. Pentru Educ. Multidim. 14, 388–405. doi: 10.18662/rrem/14.1/525

[ref45] Mikó S.-M. Hatos A. (2024). Navigating aspirations: understanding what drives Romanian adolescents' career choices. Rev. Rom. Educ. Multidimens. 16, 466–496. doi: 10.18662/rrem/16.2/867

[ref46] Morar S. M. (2024). Modelarea aspirațiilor de status la adolescenți prin scoruri de prestigiu ocupațional: concluziile unor cercetări sociologice în școlile din județul Bihor Oradea: Școala Doctorală de Sociologie, Universitatea din Oradea. Available online at: https://socioumane.ro/2024/11/19/modelarea-aspiratiilor-de-status-la-adolescenti-prin-scoruri-de-prestigiu-ocupational-concluziile-unor-cercetari-sociologice-in-scolile-din-judetul-bihor/ [Accessed March 1, 2025].

[ref47] Muntean L. M. Nireștean A. Mărușteri M. Sima-Comaniciu A. Lukacs E. Occupational stress and personality in medical doctors from Romania Healthcare 2022 MDPI:1612 doi: 10.3390/healthcare10091612 10 PMID: 36141224 PMC9498482

[ref48] Negrea A. Bekesi C. (2020). The unprecedented disruption of the coronavirus pandemic to the economy and foreign trade of the Bihor county. Ann. Univ. Oradea Econ. Sci. Ser. 29.

[ref49] OCDE (1998). Human capital investment: An international comparison. Paris, France: OECD.

[ref50] O'malley B. Y. Loomis C. Dimakos C. Lamont S. L. Singh G. Pelletier J. . (2021). I feel quite hopeful that my future is still going to be okay":" educational aspirations during COVID-19. J. Educ. Learn. 10, 78–86. doi: 10.5539/jel.v10n4p78

[ref51] Osipow S. H. Carney C. G. Barak A. (1976). A scale of educational-vocational undecidedness: a typological approach. J. Vocat. Behav. 9, 233–243. doi: 10.1016/0001-8791(76)90081-6

[ref52] Petru-Cristian N. (2024). The Nexus of proximity, talent, and opportunities: examining the challenges and prospects for young talent in Romania’s higher education context.

[ref53] Pierce M. Bai Y. Taxiarchi V. Hugh-Jones S. Abel K. M. Patalay P. . (2025). Understanding drivers of recent trends in young people’s mental health–technical report

[ref54] Qureshi N. Malik M. A. Hassan B. (2021). Students’ career aspirations and choices: comparison of grade 8 public and private school students. Int. Rev. Basic Appl. Sci. 9, 236–244.

[ref55] Rajasekaran S. Casap L. (2022) Moldova–digital education Readiness ASSESSMENT 2021-22. ASSESSMENT, 2021, 22

[ref56] Ramaiya A. Chandra-Mouli V. Both R. Gottert A. Guglielmi S. Beckwith S. . (2023). Assessing the health, social, educational and economic impact of the COVID-19 pandemic on adolescents in low- and middle-income countries: a rapid review of the literature. Sexual Reprod. Health Matters 31:2187170. doi: 10.1080/26410397.2023.2187170, PMID: 36987980 PMC10062253

[ref57] Readiness C. OECD. (2024). Teenage career uncertainty: why it matters and how to reduce it. Available online at: https://www.oecd.org/content/dam/oecd/en/publications/reports/2024/09/teenage-career-uncertainty_63c29ae4/e89c3da9-en.pdf#:~:text=PISA%20shows%20that%20two,associated%20with%20lower%20levels%20of [Accessed February 24, 2025]

[ref58] Roman M. Vasilescu M. D. (2016). Explaining the migration intentions of Romanian youth: are teenagers different. Rom. Stat. Rev. 64, 69–86.

[ref59] Romania-Insider (2022). School starts in Romania for around 2.8 mln students, with new learning models and COVID-19 prevention rules. Available online at: https://www.romania-insider.com/school-starts-romania-covid-safety-rules-2020#:~:text=School%20starts%20in%20Romania%20for,million%20preschoolers%20and%20students [Accessed February 24, 2025].

[ref60] Roșioară A.-I. Năsui B. A. Ciuciuc N. Sîrbu D. M. Curșeu D. Pop A. L. . (2024). Status of healthy choices, attitudes and health education of children and young people in Romania—a literature review. Medicina 60:725. doi: 10.3390/medicina60050725, PMID: 38792908 PMC11123286

[ref61] Santos P. J. Ferreira J. A. Gonçalves C. M. (2014). Indecisiveness and career indecision: a test of a theoretical model. J. Vocat. Behav. 85, 106–114. doi: 10.1016/j.jvb.2014.05.004

[ref62] Savickas M. L. (1997). Career adaptability: an integrative construct for life-span, life-space theory. Career Dev. Q. 45, 247–259. doi: 10.1002/j.2161-0045.1997.tb00469.x

[ref63] Savu C. Armaș I. Burcea M. Dobre D. (2023). Behind the scenes of the healthcare COVID-19 pandemic crisis: potential affecting factors of healthcare work sustainability in Romania during 2020–2022. Front. Psychol. 14:1179803. doi: 10.3389/fpsyt.2023.1179803, PMID: 37324811 PMC10267456

[ref64] Shmis T. Sava A. Teixeira J. E. N. Patrinos H. A. (2020). Response to COVID-19 in Europe and Central Asia. World Bank Group Education, Available online at: https://thedocs.worldbank.org/en/doc/862141592835804882-0090022020/original/ECAEducationResponseNotev9final.pdf [Accessed March 10, 2025].

[ref65] Siegel L. (2024). IMPACTS OF CONTEMPORARY CRISES ON THE EU ECONOMY: RETHINKING OUR APPROACH TO POLICYMAKING. Scientific papers of Silesian University of Technology. Organization & Management/Zeszyty Naukowe Politechniki Slaskiej. Seria Organizacji i Zarzadzanie 2024, 487–497. doi: 10.29119/1641-3466.2024.210.32

[ref9003] Stănculescu M. S. Marin A. M. (2022). Health-related effects at the outbreak of covid-19 pandemic in vulnerable communities of romania. Calitatea Vieții, 33, 38–55. doi: 10.46841/RCV.2022.01.03

[ref66] Stiftung F. E. (2023). Evoluția speranței de viață în timpul și ulterior pandemiei COVID 19. Available online at: https://monitorsocial.ro/indicator/1686/ [Accessed March 10, 2025].

[ref67] Strohmeier D. Gradinger P. Yanagida T. (2024). Adolescents' digital career aspirations: evidence for gendered pathways in a digital future. J. Adolesc. 96, 526–538. doi: 10.1002/jad.12258, PMID: 37811971

[ref68] Swanson N. W. Duncan N. T. (2021). Understanding the “new Normal”: the internationalization of education and study abroad during the COVID-19 era. Susan Bulkeley Butler Center for leadership excellence and ADVANCE Purdue center for faculty success working paper series, 4, 33–55.

[ref69] Tran T. Hoang A.-D. Nguyen Y.-C. Nguyen L.-C. Ta N.-T. Pham Q.-H. . (2020). Toward sustainable learning during school suspension: socioeconomic, occupational aspirations, and learning behavior of Vietnamese students during COVID-19. Sustainability 12:4195. doi: 10.3390/su12104195

[ref70] UNECE (2022). UNESCO monitors Covid-19 impact on education: a comparative perspective for the UNECE region.

[ref71] UNICEF (2021). COVID-19: schools for more than 168 million children globally have been completely closed for almost a full year [press release]. Available online at: https://www.unicef.org/romania/press-releases/covid-19-schools-more-168-million-children-globally-have-been-completely-closed#:~:text=According%20to%20UNESCO%20data%2C%20between,and%20partially%20closed%2049%20days [Accessed September 10, 2025].

[ref72] Union, E. (2024). In-depth review 2024. Romania. Available online at: https://economy-finance.ec.europa.eu/publications/depth-review-2024-romania_en [Accessed October 05, 2025].

[ref73] Von Soest T. Kozák M. Rodríguez-Cano R. Fluit S. Cortés-García L. Ulset V. S. . (2022). Adolescents’ psychosocial well-being one year after the outbreak of the COVID-19 pandemic in Norway. Nat. Hum. Behav. 6, 217–228. doi: 10.1038/s41562-021-01255-w, PMID: 35058644

[ref74] Wahyuningsih D. Wibowo M. Purwanto E. Mulawarman M. (2023). Career decision-making self-efficacy and its implications in high school students during the covid-19 pandemic. Health Educ. Health Promot. 11, 153–158. doi: 10.58209/hehp.11.1.153

[ref75] Wang M.-T. Del Toro J. Henry D. A. Scanlon C. L. Schall J. D. (2024a). Family resilience during the COVID-19 onset: a daily-diary inquiry into parental employment status, parent–adolescent relationships, and well-being. Dev. Psychopathol. 36, 312–324. doi: 10.1017/S0954579422001213, PMID: 36484143

[ref76] Wang Y. Liu D. Li C. (2024b). Mapping the foundations and evolution of career aspiration research: a bibliometric analysis. Career Dev. Int. 29, 481–493. doi: 10.1108/CDI-08-2023-0296

[ref77] Xu H. (2020). Career indecision profile–short: reliability and validity among employees and measurement invariance across students and employees. J. Career Assess. 28, 91–108. doi: 10.1177/1069072719831975

[ref78] Xu H. Tracey T. J. G. (2015). Career decision ambiguity tolerance scale: construction and initial validations. J. Vocat. Behav. 88, 1–9. doi: 10.1016/j.jvb.2015.01.006

[ref79] Yarde J. Shao X. Anders J. Cullinane C. De Gennaro A. Early E. . 2022. Future plans and aspirations. UCL Centre for Education Policy and Equalising Opportunities.

[ref80] Zhang R. Pei J. Wang Y. Wang L. Yeerjiang Y. Gao H. . (2022). COVID-19 outbreak improves attractiveness of medical careers in Chinese senior high school students. BMC Med. Educ. 22:241. doi: 10.1186/s12909-022-03309-7, PMID: 35379234 PMC8978502

